# Fibre morphology, intramyocellular lipid content and 3D capillary architecture in human postural, respiratory and locomotor muscles in type 2 diabetes mellitus

**DOI:** 10.1007/s00418-026-02477-7

**Published:** 2026-04-06

**Authors:** Nataša Pollak, Jiří Janáček, František Saudek, Erika Cvetko, Barbora Radochová, Armin Alibegović, Chiedozie Kenneth Ugwoke, Davide Alessandro Basello, Žiga Šink, Luka Pušnik, Rok Tit Tomazin, Nejc Umek

**Affiliations:** 1 Institute of Anatomy, Faculty of Medicine, University of Ljubljana, Korytkova 2, 1000 Ljubljana, Slovenia; 2 Laboratory of Advanced Microscopy and Data Analyses, Institute of Physiology, The Czech Academy of Sciences, Prague, Czech Republic; 3 Diabetes Centre, Institute for Clinical and Experimental Medicine, Prague, Czech Republic; 4 Institute of Forensic Medicine, Faculty of Medicine, University of Ljubljana, Ljubljana, Slovenia

**Keywords:** Type 2 diabetes mellitus, Skeletal muscle, Myosin heavy chain isoforms, Intramyocellular lipid, 3D capillary morphometry, Metabolic myopathy

## Abstract

In type 2 diabetes mellitus (T2DM), skeletal muscle is a major site of metabolic and microvascular dysfunction, yet most human data derive from large locomotor muscles, whereas postural and respiratory muscles remain less well characterised. We examined whether T2DM alters fibre morphology, intramyocellular lipid (IMCL) content, and three-dimensional (3D) capillary architecture across functionally distinct muscles. Postural (splenius capitis [SC]), respiratory (diaphragm [DIA]; external intercostal [EXT]), and locomotor (vastus lateralis [VL]) muscles from adult male individuals (T2DM versus control, *n* = 24/group) were sampled < 24 h post-mortem. Analysis included myosin heavy chain fibre typing, Sudan Black B intramyocellular lipid (IMCL) quantification, and 3D capillary morphometry (length, tortuosity, anisotropy, branching density). Groups were age-matched (T2DM 70.8 ± 7.4 versus 69.7 ± 11.8 years; *p* = 0.684), but body mass index (BMI) was higher in T2DM (31.9 ± 4.7 versus 24.8 ± 2.7 kg m^−2^; *p* < 0.0001). Fibre-type profiles were similar, except for elevated 2a/2x hybrids in T2DM VL (*p* = 0.014). Mean fibre diameters were preserved, though type 1 fibres were larger in T2DM SC (*p* = 0.0238). IMCL was higher in T2DM SC and EXT (*p* < 0.05), with non-significant differences in VL and DIA. Type 1 and 2a fibres had higher IMCL than glycolytic fibres, with no group-by-fibre-type interaction. BMI strongly predicted VL IMCL (*p* < 0.0001), while age was negatively associated with IMCL in respiratory muscles (*p* ≤ 0.05). Capillary length per fibre volume was selectively reduced in DIA (*p* = 0.0115); other indices were preserved, except for higher anisotropy in EXT (*p* = 0.0495). Overall, these functionally diverse muscles showed subtle muscle-specific remodelling in T2DM, with adiposity-linked IMCL accumulation and reduced DIA capillary supply, although findings should be interpreted in the context of the post-mortem study.

**Supplementary Information:**

The online version contains supplementary material available at 10.1007/s00418-026-02477-7.

## Introduction

Type 2 diabetes mellitus (T2DM) is associated with impaired skeletal muscle oxidative metabolism, including reduced expression or abundance of oxidative phosphorylation components and reduced insulin-stimulated non-oxidative glucose disposal, which together contribute to dysglycaemia (Patti et al. 2003; Yokoyama et al. 2008; Öhman et al. 2021). Skeletal muscle is the principal site of insulin-stimulated glucose disposal in humans and comprises roughly 30–40% of adult body mass, underscoring its central role in systemic insulin resistance (Baron et al. 1988; Janssen et al. 2000). In T2DM, changes in fibre-type composition and oxidative capacity, mitochondrial dysfunction, and impaired microvascular perfusion collectively contribute to reduced aerobic metabolism and insulin responsiveness (Oberbach et al. 2006; Park et al. 2020; Öhman et al. 2021). However, most human data are derived from superficial locomotor muscles such as the vastus lateralis, whereas postural and respiratory muscles that sustain continuous low-intensity activity for posture and breathing remain comparatively understudied.

In locomotor muscles, obesity and T2DM have often been associated with a relative shift away from slow oxidative fibres and reduced oxidative enzyme activity, although findings vary across cohorts (He et al. 2001; Oberbach et al. 2006). Conversely, ageing is often characterised by preferential atrophy and loss of type II fibres, which can increase the relative representation of slow oxidative fibres, although the extent of this remodelling varies by muscle (Lexell et al. 1988; Frontera et al. 2000). Intermuscular heterogeneity is considerable; fibre-type proportions differ markedly between neck and limb muscles even within the same individual (Vikne et al. 2012; Cornwall and Kennedy 2015). In obesity and T2DM, chronic metabolic overload further promotes the accumulation of intramyocellular lipid (IMCL), a critical energy reservoir and lipid buffer (da Silva Rosa et al. 2020). Seminal proton magnetic resonance sprectroscopy (1H-MRS) studies reported an inverse association between IMCL and insulin sensitivity in humans (Krssak et al. 1999), and subsequent work implicated lipid-derived intermediates, including diacylglycerols and ceramides and their intracellular localisation, in impaired insulin signalling and mitochondrial function (Szendroedi et al. 2014; Perreault et al. 2018).

The skeletal muscle capillary network is essential for oxygen and substrate delivery and is affected by obesity, ageing, and T2DM, although human findings remain variable across studies and populations (Prior et al. 2015; Park et al. 2020). Early clamp studies associated reduced capillary density and a lower proportion of slow oxidative (type 1) fibres with insulin resistance (Lillioja et al. 1987), whereas later work demonstrated that insulin-mediated microvascular recruitment is blunted in obesity and T2DM (Clerk et al. 2006; Park et al. 2020). Ageing and T2DM can also impair microvascular function, including endothelial glycocalyx alterations, with effects that depend on muscle type and physical activity level (Groen et al. 2014; Prior et al. 2015; Bosutti et al. 2015). Conventional two-dimensional capillary profile counts are sensitive to section orientation and fibre size, which can bias estimates of capillary supply in architecturally complex muscles. Modern three-dimensional confocal imaging with vector-based reconstruction enables direct quantification of capillary length and network geometry (tortuosity, anisotropy, and branching density) and permits correction for axial shrinkage in thick sections, providing a less biased representation of microvascular architecture (Čebašek et al. 2010; Janáček et al. 2011, 2012).

Against this background, we analysed autopsy samples from four functionally distinct muscles (postural, respiratory and locomotor), from individuals with and without T2DM. These muscles differ in metabolic phenotype and habitual functional demands, spanning from near-continuous respiratory or postural activity to intermittent locomotion (Mizuno 1991; Pollak et al. 2025). Using myosin heavy chain (MyHC)-based fibre typing, Sudan Black B histochemistry and three-dimensional (3D) confocal morphometry, we sought to determine whether T2DM is associated with muscle-specific alterations in fibre-type composition, fibre size, IMCL content and capillary network geometry, and whether the structural hierarchy among postural, respiratory and locomotor muscles is preserved in T2DM.

## Methods

### Study design

This comparative study was conducted on human skeletal muscle samples obtained post-mortem from individuals with T2DM and age-matched non-diabetic controls (*n* = 24 per group). Four functionally distinct skeletal muscles were analysed: splenius capitis (SC) as a predominantly postural cranio-cervical muscle, the respiratory muscles external intercostal (EXT) and diaphragm (DIA), and vastus lateralis (VL) as a predominantly locomotor lower limb muscle (Zupančič et al. 2023; Pollak et al. 2025). Muscle samples were collected from each individual within 24 h post-mortem during routine autopsies at the Institute of Forensic Medicine, University of Ljubljana. Cause-of-death data were available for all 48 cases. To reduce the risk of deductive identification in this relatively small forensic cohort, causes of death are reported here in broad categories. In the control cohort, deaths were due to traumatic causes (21/24) or sudden cardiovascular events (3/24). In the T2DM cohort, deaths were predominantly due to acute cardiovascular, thromboembolic, or cerebrovascular causes (20/24), with the remaining cases due to traumatic causes (4/24). Exclusion criteria included type 1 diabetes mellitus; physician-diagnosed systemic rheumatic disease; prolonged pre-mortem immobilization; prior trauma, surgery, or local corticosteroid injection at any planned sampling site; current or recent systemic glucocorticoid therapy, systemic hormonal therapy, heritable neuromuscular disorders, active malignancy, prior radiotherapy or cytotoxic chemotherapy; and any macroscopic pathology or evident post-mortem degradation of the sampled muscles at autopsy. Detailed agonal duration and peri-mortem medication data were not available in a standardised form suitable for covariate analysis. Only male individuals were included to reduce biological heterogeneity and to avoid sex as an additional effect modifier in this multi-muscle morphometric study, because standardised information on menopausal status, hormone therapy, and other sex-related covariates was not available for all cases in the post-mortem records. Long-term glycaemic control in the T2DM group and normoglycaemia in the control group were evaluated using post-mortem HbA1c. This marker remains stable after death, providing an objective assessment of ante-mortem glycaemic status for all subjects. Medical records indicated a T2DM duration of at least 10 years in all affected individuals, and that glucose-lowering management consisted of oral antihyperglycaemic agents administered as monotherapy or combination therapy (most commonly metformin, sulfonylureas and/or DPP-4 inhibitors). Peri-mortem medications administered during terminal care or resuscitation were not available in a standardised form across cases and were therefore not analysed as covariates. All procedures were reviewed and approved by the National Medical Ethics Committee of the Republic of Slovenia (permit nos. 0120–536/2019/4 and 0120/536/2019/7) and were conducted in accordance with the Declaration of Helsinki.

### Muscle tissue sampling

Muscle samples were obtained from standardised anatomical sites: SC at the mid-portion (C4 level); EXT at the sixth intercostal space (midclavicular line); DIA at the costal portion (midclavicular line); and VL at the distal third of the thigh. Each sample (≈1 cm^3^) was rapidly frozen in liquid nitrogen and stored at −80 °C until further processing. Serial transverse cryosections were prepared using a Leica CM1950 cryostat (Leica Microsystems GmbH, Wetzlar, Germany). Thin 10-µm sections were used for histochemistry and fibre typing, whereas adjacent thick 100-µm sections were prepared for three-dimensional (3D) capillary analysis.

### Histochemistry: intramyocellular lipids

Neutral lipids were visualised using Sudan Black B staining (Sigma-Aldrich Corp, St. Louis, MO, USA) on 10-µm cryosections. Serial sections from the same sample were analysed to align fibre type and lipid measurements. Sections were equilibrated to room temperature, rinsed briefly in 70% ethanol, stained for 60 min in saturated Sudan Black B solution prepared in 70% ethanol, rinsed in tap water and mounted in glycerol-gelatin. The IMCL index was calculated as the percentage of fibre area occupied by Sudan Black-positive lipid droplets, providing a comparative two-dimensional morphometric estimate of bulk intracellular lipid storage.

### Immunohistochemistry: myosin heavy chain isoform expression

Serial 10-µm transverse sections were incubated with normal rabbit serum (1:40 in phosphate-buffered saline [PBS] containing 0.5% bovine serum albumin [BSA]) to block non-specific binding, followed by incubation with monoclonal antibodies against MyHC isoforms: BA-D5 (MyHC-1), SC-71 (MyHC-2a) and 6H1 (MyHC-2x) (Developmental Studies Hybridoma Bank, Iowa City, IA, USA), each diluted 1:100 in PBS (pH 7.4). Specificity of these clones for adult MyHC isoforms and their use for fibre typing have been established by published biochemical and histochemical validation studies (Schiaffino et al. 1989; Lucas et al. 2000; Smerdu and Soukup 2008; Bloemberg and Quadrilatero 2012). Immunoreactivity was visualised using a diaminobenzidine (DAB)-based peroxidase detection system (Novolink Polymer Detection System RE7150-K; Leica Biosystems, Newcastle, UK) according to the manufacturer’s instructions. As a procedural negative control, the primary antibody was omitted; no horseradish peroxidase (HRP)/DAB-derived reaction product was observed (Schiaffino and Reggiani 2011). Fibres were classified as type 1, type 2a, type 2x, and hybrid type 1/2a and type 2a/2x by matching the same fibre across the three serial MyHC sections in a fixed order (BA-D5, SC-71, 6H1) and interpreting relative staining intensity patterns, consistent with established MyHC-based fibre typing conventions. Hybrid fibres were defined by concomitant positivity on two antibodies in the serial sections, specifically BA D5 plus SC 71 for type 1/2a and SC 71 plus 6H1 for type 2a/2x (Schiaffino and Reggiani 2011).

### Light microscopy and image analysis

Images of MyHC-stained serial sections were acquired in brightfield on a Nikon Eclipse 80i microscope (Nikon Corporation, Tokyo, Japan) using a 20× Plan Fluor objective (numerical aperture 0.50) equipped with a KERN ODC 841 digital camera (KERN & SOHN GmbH, Balingen, Germany) and VIS Pro KERN OXM 902 software. Illumination, aperture, and exposure settings were standardised within each imaging batch to ensure reproducible conditions. High-resolution fields (5440 × 3648 pixels) were sampled systematically, with at least three randomly selected regions per muscle, yielding a minimum of 100 analysed fibres per muscle (total area: ~8.3 × 10^5^ μm^2^) (Supplementary Table S1). Images were saved as 24-bit RGB TIFF files (8 bits per colour channel). Image analysis was performed in Ellipse 2.081 (ViDiTo, Košice, Slovakia). Fibre-type classification was conducted using the dedicated software developed by Karen et al. (Karen et al. 2009), which semi-automatically aligns serial sections and assigns fibre types according to myosin heavy chain expression profiles. For each muscle, fibre-type proportions (%) and fibre diameter, defined as the minimal Feret diameter, were calculated, and a pooled whole-muscle mean fibre diameter was computed for each subject by averaging the minimal Feret diameter across all typed fibres within that muscle. The IMCL index was determined as the percentage of the muscle fibre cross-sectional area occupied by Sudan Black B-positive lipid droplets. IMCL was quantified in the same fibres used for MyHC-based fibre typing, so the number of fibres contributing to each fibre-type IMCL estimate per participant reflected the underlying fibre-type proportions. All imaging and analyses were performed by a single trained evaluator blinded to group.

### 3D capillary network labelling

Thick (100 µm) transverse sections were washed in cold PBS with 0.1% Triton X-100 (PBST; Sigma-Aldrich Corp, St. Louis, MO, USA) and fixed at 4 °C in 7% formaldehyde with 0.1% glutaraldehyde in PBST. After PBST washes, antigen retrieval was performed with 0.2% proteinase K (Fermentas, Waltham, MA, USA) (0.5 M Tris, pH 8.0, with EDTA) for 5 min at 37 °C, followed by further PBST washes. The basal lamina was labelled overnight at 4 °C with rabbit anti-collagen IV polyclonal antibody (1:200; ab6586; Abcam, Cambridge, UK), followed by Alexa Fluor 594-conjugated goat anti-rabbit IgG secondary antibody (1:500; A-11012; Invitrogen, Thermo Fisher Scientific, Waltham, MA, USA). Endothelial cells were labelled overnight at 4 °C with fluorescein-labelled Griffonia simplicifolia lectin I (1:300; FL-1101; Vector Laboratories, Newark, CA, USA) and mouse monoclonal antibody F8/86 anti-von Willebrand factor (1:1000; M0616; Dako, Agilent Technologies, Glostrup, Denmark), followed by Alexa Fluor 488-conjugated goat anti-mouse IgG secondary antibody (1:500; A11001; Invitrogen, Thermo Fisher Scientific, Waltham, MA, USA). Marker specificity was supported by the expected anatomical localisation of collagen IV to the basal lamina and of lectin and von Willebrand factor to the endothelium, and by established use of this marker combination for 3D confocal quantification of skeletal muscle microvasculature (Kubínová et al. 2001; Janáček et al. 2011). Sections were mounted in ProLong™ Gold Antifade Mountant (P36930; Thermo Fisher Scientific, Waltham, MA, USA). This thick-section staining workflow was based on previously established human skeletal muscle 3D capillarity protocols in 100-µm sections, and all samples in the present study were processed using the same section thickness, permeabilization, antigen retrieval, incubation times, and reagent concentrations across muscles and groups (Janáček et al. 2011). Negative controls omitting primary antibodies showed no specific immunofluorescent signal.

### Confocal imaging and 3D reconstruction

Image stacks were acquired using a Leica STELLARIS 8 confocal microscope (Leica Microsystems GmbH, Wetzlar, Germany) with a 40×/1.1 water-immersion objective. Z-stacks were obtained with 1-µm optical steps at a resolution of 512 × 512 pixels (pixel size 0.76 µm). A linear laser power Z-compensation was utilised to avoid loss of signal owing to sample thickness. To reduce depth-related attenuation bias, image stacks were acquired using the same optical step size and batch-matched acquisition settings, and segmented capillary reconstructions were subsequently reviewed and manually refined in Tracer3D by a blinded evaluator. Sequential excitation at 488 nm (detection: 498–585 nm) and 570 nm (detection: 590–700 nm) was used to avoid channel crosstalk. Excitation was provided by a white light laser, and two HyD X detectors, set to operate in photon-counting mode, were used for detection. For each muscle, five randomly selected fields of view (387.5 × 387.5 µm) containing at least 100 fibres in total were analysed. Image stacks were processed in Ellipse 2.081 (ViDiTo, Košice, Slovakia). Z-axis deformation was corrected by axial calibration (Janáček et al. 2011). After segmentation, binary images were skeletonised using the six-pass Palágyi algorithm and vectorised into 5-µm line segments, followed by manual refinement in Tracer3D (Cvetko et al. 2013) using a Phantom Omni haptic device (3D Systems, Rock Hill, SC, USA). Fibre contours were traced on four planes to calculate fibre diameter, surface area and volume. Quantitative parameters included capillary length per fibre length (LL), per fibre surface area (LSf), and per fibre or muscle volume (LVf, LVm) (Janáček et al. 2011; Cvetko et al. 2013). Mean capillary length (MeanCap) was computed as two-thirds of the total capillary length per unit volume divided by the number of branching points per unit volume, i.e. $MeanCap=\frac{2}{3}\frac{{L}_{V}}{{N}_{V}}$, where *L*_V_ is capillary length density, and *N*_V_ is branching-point density (Janáček et al. 2011). Tortuosity was expressed as the ratio of the sum of exterior angles to the total capillary length anisotropy as the ratio of the principal eigenvalues of the structural tensor, and branching density (Br_dens) as branch points per muscle volume (Cvetko et al. 2013; Eržen et al. 2018). All analyses were performed by the same evaluator, blinded to group.

### Statistical analysis

All analyses were performed in Python (Statsmodels 0.14; Python Software Foundation, Wilmington, DE, USA) and GraphPad Prism 10 (GraphPad Software, LLC, San Diego, CA, USA). Normality of distributions and model residuals was assessed using the Shapiro–Wilk and Jarque–Bera tests. Between-group differences in demographic variables were evaluated with independent-samples *t*-tests. Multivariable models were prespecified and adjusted for age and body mass index (BMI). For capillary parameters (LVf, LVm, LL, LSf, MeanCap, Br_dens, tortuosity, anisotropy), linear mixed-effects models were fitted with subject as a random intercept to account for repeated fields of view within individuals. Models were fitted separately for each muscle, with fixed effects for group, age, and BMI, and the adjusted group effect was reported as β with 95% confidence intervals (CIs) and two-sided *p*-values. Sensitivity analyses included quadratic age (age^2^) and group-by-age terms. For LVf, an additional model included mean fibre diameter as a covariate. For fibre-level outcomes (fibre-type proportions, fibre diameter, and IMCL index), subject-level means were calculated for each fibre type (1, 2a, 2x, 1/2a, and 2a/2x) within each muscle. These means were compared using two-way analysis of variance (ANOVA) (group × fibre type) with Tukey post hoc tests. Because all four muscles were sampled from the same individuals, within-subject comparisons across muscles, including fibre-type proportions, mean fibre diameter, and IMCL index, were assessed using paired *t*-tests and a repeated-measures mixed model of the form outcome ~ group × muscle + age + BMI + (1|subject) to estimate muscle-specific group effects and group-by-muscle interactions. Within the T2DM subgroup, associations between HbA1c and structural outcomes were examined using linear models adjusted for age and BMI. All tests were two-sided with *α* = 0.05, and results are presented as mean ± standard deviation (SD) or adjusted regression coefficients (β) with 95% confidence intervals and corresponding *p*-values.

## Results

### Study groups

Age did not differ significantly between groups (T2DM 70.8 ± 7.4 versus controls 69.7 ± 11.8 years; *p* = 0.684), whereas BMI was higher in T2DM (31.9 ± 4.7 versus 24.8 ± 2.7 kg m^−2^; *p* < 0.0001). The mean duration of T2DM was 16.5 ± 5.1 years. In the control group, all subjects had HbA1c within the normal range (< 5.7% [< 39 mmol mol^−1^]), while in the T2DM group, mean HbA1c was 6.9 ± 1.1% (51.9 ± 12.4 mmol mol^−1^); 4 of 24 individuals (17%) had HbA1c > 8.0%.

### Muscle fibre morphology

**Fig. 1:**
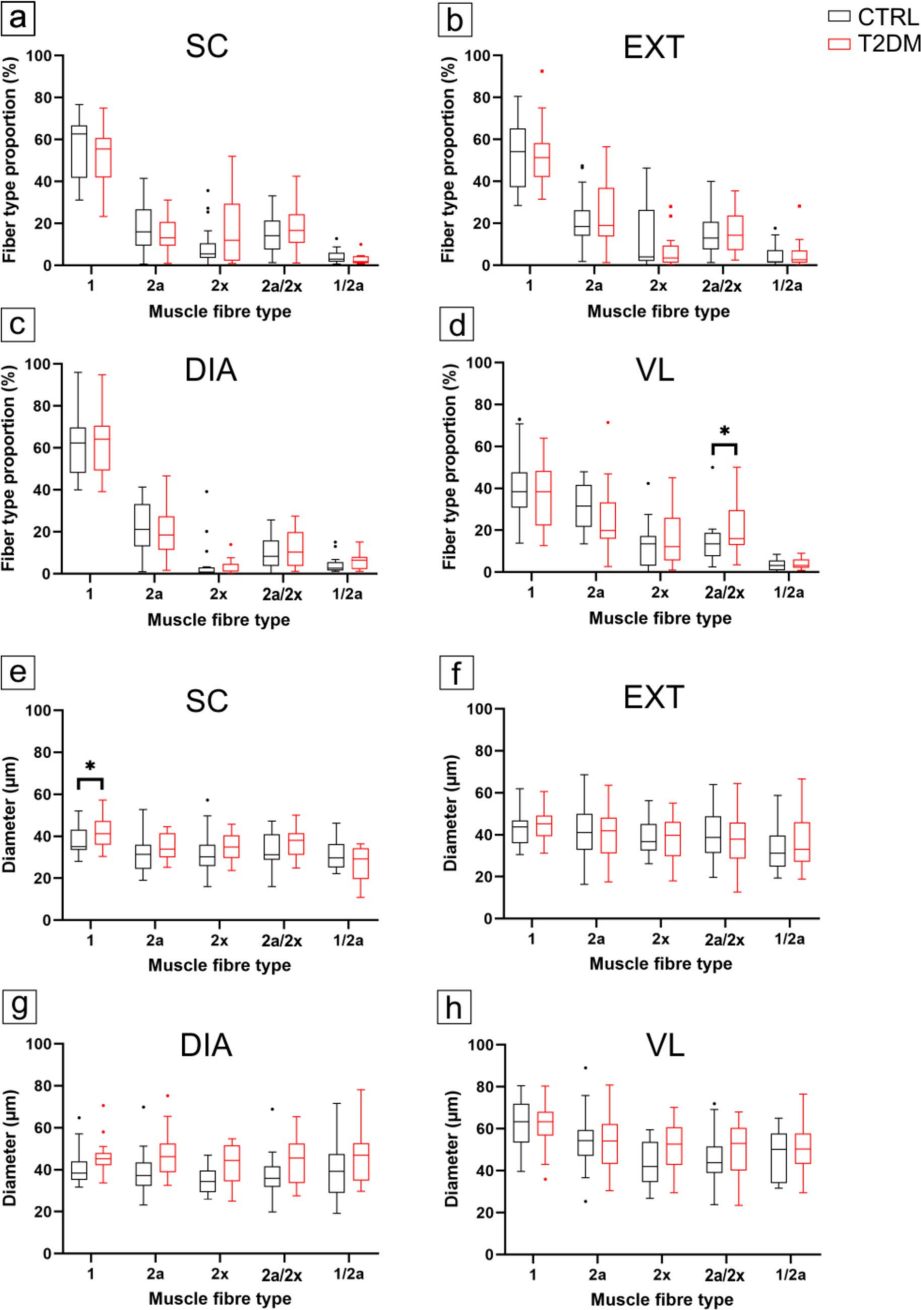
Fibre-type proportions and fibre diameter in control and T2DM. Fibre-type proportions (**a**–**d**) and fibre diameter (**e**–**h**) in splenius capitis (SC; **a**, **e**), external intercostal muscle (EXT; **b**, **f**), diaphragm (DIA; **c**, **g**) and vastus lateralis muscle (VL; **d**, **h**) in control (black) and T2DM (red) subjects. Boxes represent the interquartile range (IQR), centre lines denote medians, whiskers indicate 1.5 × IQR (Tukey) and points denote outliers. Asterisks (*) above the bars denote *p* < 0.05. CTRL, control; T2DM, type 2 diabetes mellitus

**Fig. 2:**
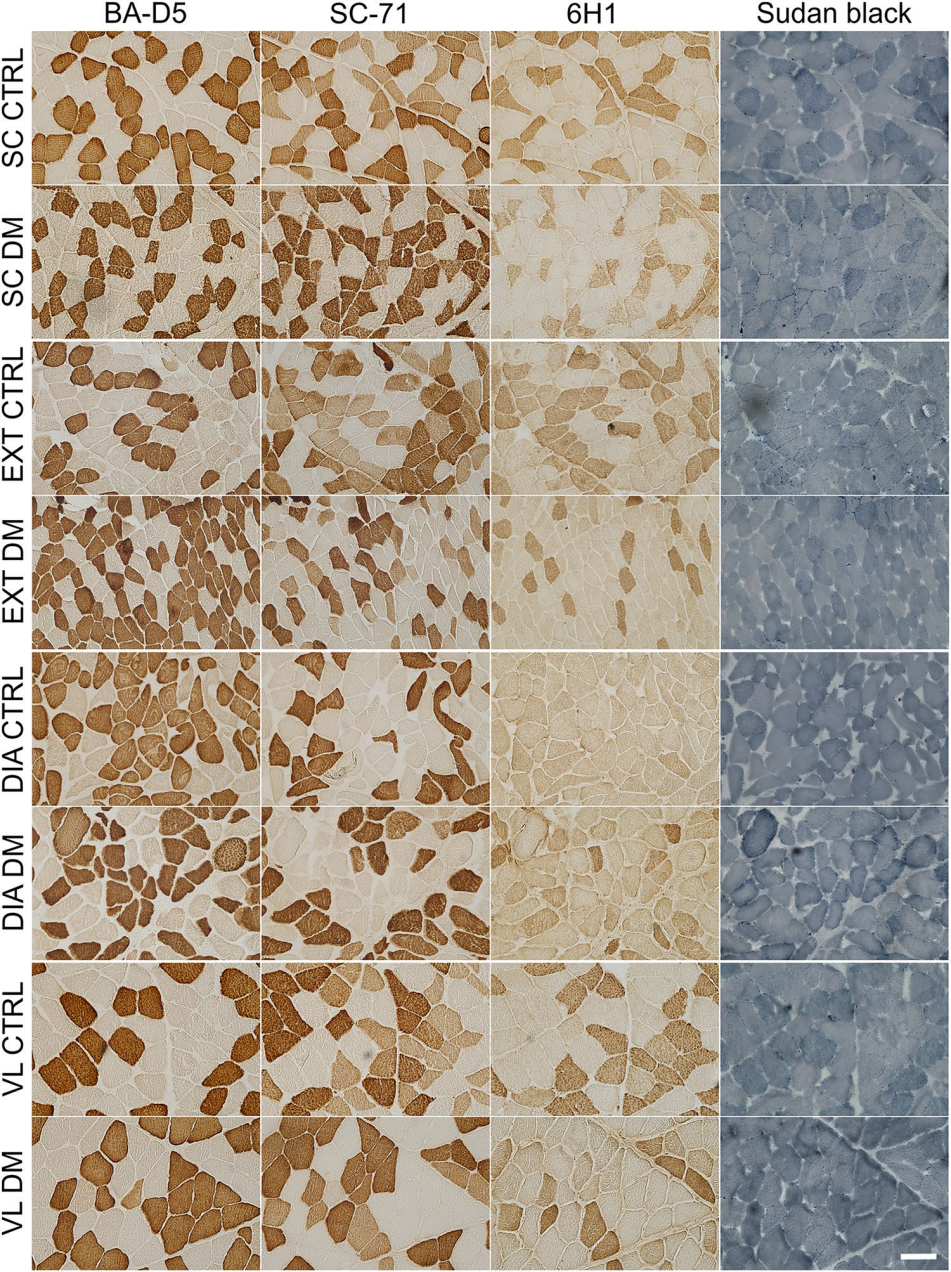
Fibre type-specific myosin heavy chain expression and intramyocellular lipid content in four human skeletal muscles from control and T2DM subjects. Expression of MyHC isoforms type 1, type 2a, type 2x, and intramyocellular lipid visualised by Sudan Black in successive transverse sections of the splenius capitis (SC), diaphragm (DIA), external intercostal (EXT) and vastus lateralis (VL) muscles. Within each group, panels are arranged from left to right as follows: BA-D5 (MyHC-1-positive fibres, including type 1 and 1/2a hybrids), SC-71 (MyHC-2a-positive fibres, including type 2a and hybrid fibres), 6H1 (MyHC-2x-positive fibres, including type 2x and 2a/2x hybrids), and Sudan Black B (IMCL, intramyocellular lipid). Fibre types and hybrids were assigned by matching fibres across serial BA-D5, SC-71 and 6H1 sections and interpreting staining intensity patterns, with hybrids defined by concomitant positivity on two antibodies, specifically BA-D5 plus SC-71 for type 1/2a and SC-71 plus 6H1 for type 2a/2x, according to established MyHC-based fibre typing conventions. Scale bar = 100 μm. DIA, diaphragm; SC, splenius capitis; EXT, external intercostal; VL, vastus lateralis; CTRL, control; DM, diabetes mellitus (type 2)

Overall, fibre-type composition and fibre diameter were largely similar between groups, with only muscle-specific differences. Fibre-type composition (Fig. 1a–d, Fig. 2) did not differ significantly between groups in SC, DIA, or EXT (all *p* ≥ 0.10). In the VL, the proportion of hybrid type 2a/2 × fibres was higher in T2DM than in controls (*p* = 0.0141), whereas all other fibre types did not differ significantly between groups. Pooled whole-muscle mean fibre diameter, calculated across fibre types, did not differ significantly between groups in any of the examined muscles, although SC displayed a borderline trend toward larger mean diameters in T2DM (*p* = 0.0502). In fibre-type-specific analyses (Fig. 1e–h), the only significant group difference was observed in the SC, where type 1 fibres were larger in T2DM than in controls (*p* = 0.0238); no other fibre types in the SC, DIA, EXT or VLs differed between groups (all *p* > 0.10). Within-subject comparisons across muscles showed clear intermuscular differences in fibre-type proportions. In both groups, SC, DIA and EXT displayed higher proportions of type 1 fibres than VL, whereas VL showed a higher proportion of total type 2 fibres (Fig. 1a–d).

Across the combined cohort, age was positively associated with fibre diameter in several fast and hybrid fibre types, including type 1/2a, 2a, 2a/2x and 2x fibres in the EXT, and type 2a, 2a/2x and 2x fibres in the VL, as well as type 2x fibres in the DIA (all *p* < 0.05), whereas no age effect was detected in any fibre type in the SC. BMI showed no independent association with fibre diameter in any muscle. Within the T2DM subgroup, HbA1c was not associated with fibre diameter except for an isolated positive association in type 2x fibres of the EXT (*p* = 0.0396).

### Intramyocellular lipids

**Fig. 3:**
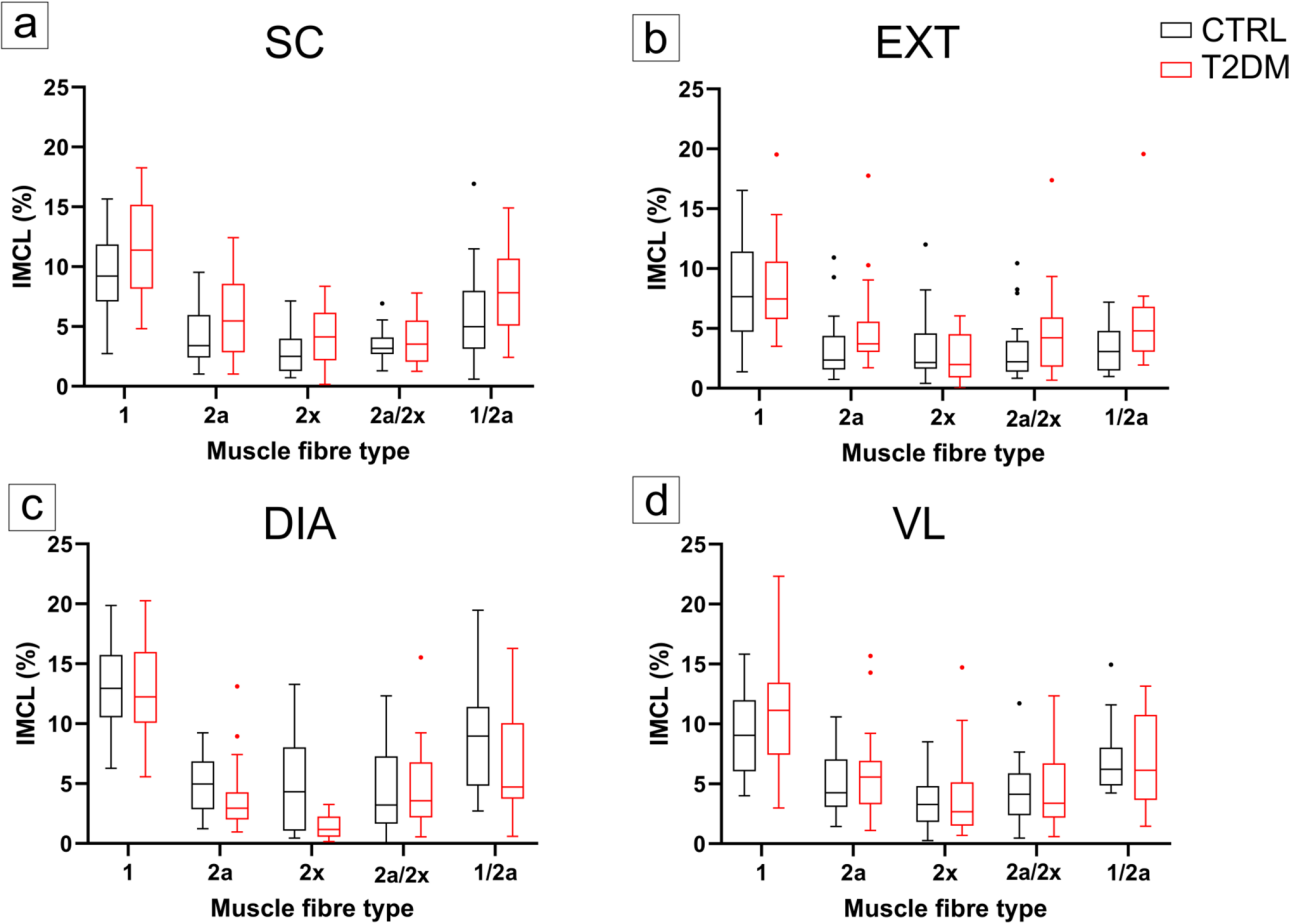
Fibre-type-specific intramyocellular lipid content (IMCL) in control and diabetic subjects. **a**–**d** IMCL (%) across fibre types in the splenius capitis (SC; **a**), external intercostal (EXT; **b**), diaphragm (DIA; **c**) and vastus lateralis (VL; **d**) muscles. Boxes represent the interquartile range; centre lines denote medians; whiskers indicate 1.5 × IQR (Tukey). Individual points represent observed values. CTRL, control (black); T2DM, type 2 diabetes mellitus (red)

The IMCL index was higher in T2DM, with significant group differences in the SC (*p* = 0.0128) and EXT (*p* = 0.0195) muscles and no significant differences in the VL (*p* = 0.111) and DIA (*p* = 0.433) (Fig. 3). Across fibre types, type 1 and 2a fibres contained more IMCL than type 2x fibres, with no group-by-fibre-type interaction.

BMI was the strongest independent predictor of IMCL, especially in VL (*β* = +0.28 percentage points per kg m^−2^, 95% CI +0.19 to +0.36, *p* < 0.0001), while age was modestly negatively associated with IMCL in DIA and EXT (*β* = −0.017 and −0.015 percentage points per year, both *p* ≤ 0.05). Within the T2DM subgroup, HbA1c showed no consistent association with IMCL in pooled models (*p* = 0.7100), and all muscle-fibre combinations were non-significant (*p* ≥ 0.10) except for a small isolated negative association in DIA 1/2a fibres (*p* = 0.0419).

### Three-dimensional capillary architecture

**Fig. 4:**
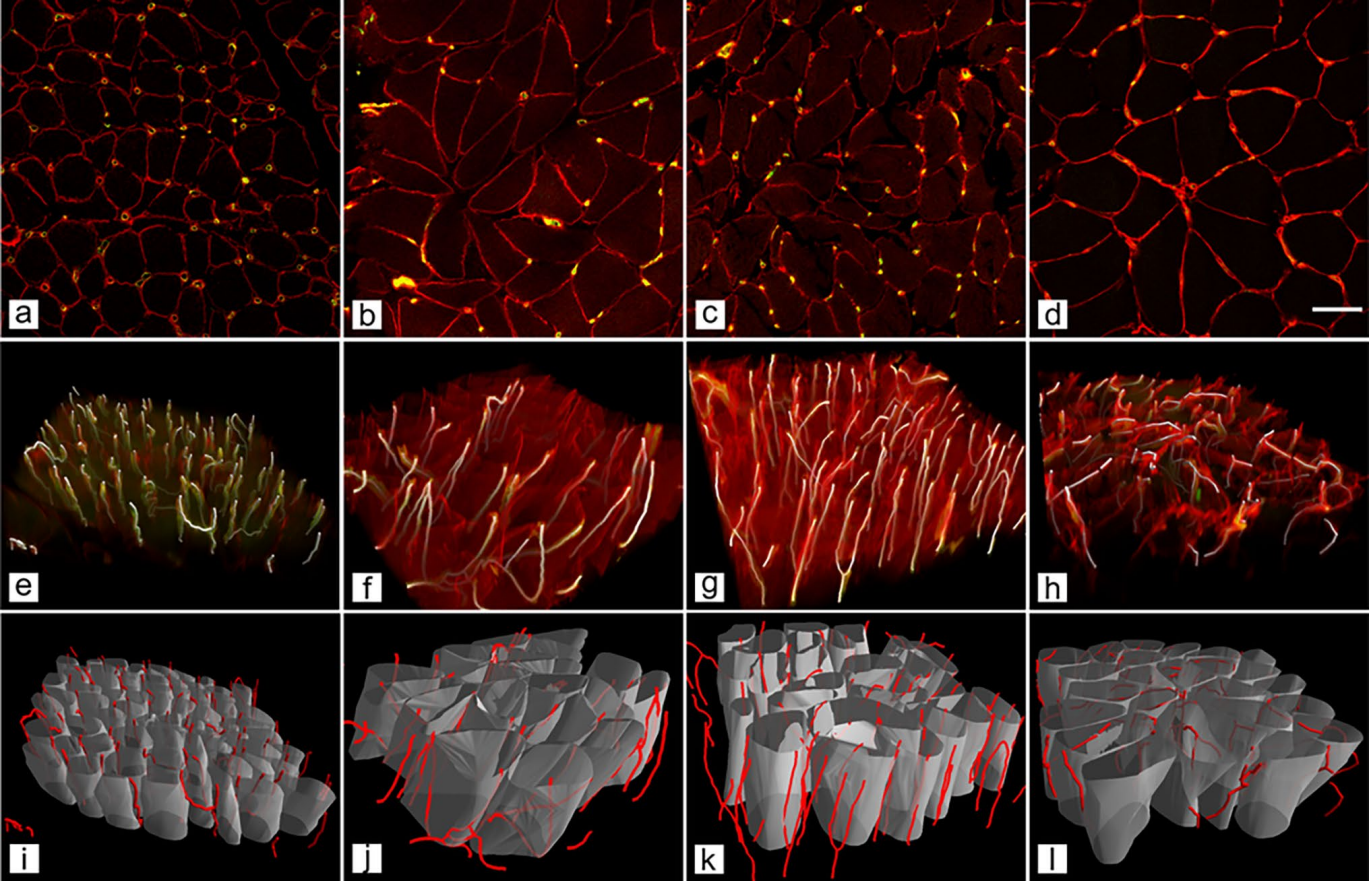
Representative confocal z-stack images and 3D reconstructions of the capillary network across four human skeletal muscles from the control group. **a**–**d** Representative single optical sections from confocal z-stacks showing merged channels from triple immunofluorescence staining: capillaries (yellow–green) and muscle fibre outlines (red). These panels illustrate the spatial distribution of capillaries relative to fibre boundaries within the image volume used for 3D analysis. **e**–**h** Volume renderings generated from the full z-stacks for the same regions, with the capillary network segmented and displayed throughout the reconstructed volume. **i**–**l** 3D renderings combining muscle fibre volumes (semi-transparent grey) with the reconstructed capillary network (red) for the same regions. Columns correspond to muscles as follows: splenius capitis (**a**, **e**, **i**), external intercostal (**b**, **f**, **j**), diaphragm (**c**, **g**, **k**) and vastus lateralis (**d**, **h**, **l**). Scale bar = 50 μm (applies to **a**–**d**)

Three-dimensional morphometry showed a selective reduction in capillary length per fibre volume (LVf) in the DIA in T2DM, with otherwise preserved capillary geometry and minor, muscle-specific differences. Descriptive values for all capillary parameters are presented in Table 1, and representative confocal z-stack images and 3D reconstructions are shown in Fig. 4.

**Table 1:** Three-dimensional capillary architecture across four human skeletal muscles in non-diabetic controls and individuals with T2DM

Parameter	Group	DIA	SC	EXT	VL
LVm (µm^−2^ × 10^−6^)	CTRL T2DM	598.75 ± 122.01 601.83 ± 193.74	415.32 ± 70.31 418.83 ± 72.34	422.02 ± 81.17 438.00 ± 91.90	368.89 ± 74.80 435.85 ± 143.04
Tortuosity (rad µm^−1^ × 10^–3^)	CTRL T2DM	23.21 ± 8.20 20.56 ± 4.97	30.78 ± 11.33 33.37 ± 12.48	26.60 ± 10.27 19.94 ± 5.16	42.12 ± 10.30 42.72 ± 10.21
Anisotropy	CTRL T2DM	2.75 ± 0.35 2.61 ± 0.62	2.50 ± 0.36 2.34 ± 0.41	2.24 ± 0.32* 2.56 ± 0.45	1.66 ± 0.36 1.61 ± 0.24
MeanCap (µm)	CTRL T2DM	373.76 ± 151.00 308.61 ± 202.80	469.86 ± 479.55 415.09 ± 264.33	330.18 ± 147.38 370.10 ± 159.52	236.91 ± 62.56 222.15 ± 73.24
Br_dens	CTRL T2DM	1.37 ± 0.49 2.04 ± 1.48	0.93 ± 0.32 0.99 ± 0.52	1.11 ± 0.37 1.08 ± 0.55	1.16 ± 0.34 1.51 ± 0.62
LVf (µm^−2^ × 10^–4^)	CTRL T2DM	20.09 ± 6.46* 17.06 ± 6.63	13.64 ± 3.86 12.58 ± 4.74	12.57 ± 4.10 13.03 ± 4.24	10.13 ± 2.80 9.72 ± 3.78
LSf (µm^−1^ × 10^–4^)	CTRL T2DM	200.29 ± 59.60 197.39 ± 68.55	126.42 ± 28.81 136.17 ± 50.30	134.78 ± 34.83 135.25 ± 32.39	145.96 ± 37.81 142.45 ± 57.78
LL (µm)	CTRL T2DM	3.39 ± 1.10 3.93 ± 1.50	1.94 ± 0.55 2.47 ± 1.28	2.47 ± 0.63 2.52 ± 0.81	3.70 ± 1.61 3.59 ± 1.69

Capillary network characteristics were estimated by the length of capillaries per volume of muscle tissue (LVm, [μm^–2^] × 10^–6^), length of capillaries per length of muscle fibres (LL), length of capillaries per fibre surface (LSf, [μm^–1^] × 10^–4^), length of capillaries per fibre volume (LVf, [μm^–2^] × 10^–4^), tortuosity ([rad μm^–1^] × 10^–3^), anisotropy, mean capillary length (MeanCap, µm); number of branching per muscle volume (Br_dens, [μm^–3^] × 10^–6^). Values are mean ± SD

*SC* splenius capitis, *DIA* diaphragm, *EXT* external intercostal, *VL* vastus lateralis, *CTRL* control, *T2DM* type 2 diabetes mellitus

*Denotes *p* < 0.05

LVf was significantly lower in DIA in T2DM than in controls (*p* = 0.0115), with a non-significant trend toward lower LVf in SC (*p* = 0.0768) and no group differences in EXT or VL (both *p* > 0.10). Anisotropy was modestly higher in EXT in T2DM (*p* = 0.0495), whereas LVm, LL, LSf, MeanCap, tortuosity and Br_dens showed no group differences (all *p* > 0.10).

In covariate analyses, age was associated with lower tortuosity in SC (*p* = 0.0140) and VL (*p* = 0.0401) and with shorter LL in VL (*p* = 0.0142). BMI was positively associated with LVf (*p* = 0.0236) and LSf (*p* = 0.0444) in DIA. Within the T2DM subgroup, HbA1c showed DIA-specific associations, being inversely related to LL (*p* = 0.0367) and positively related to MeanCap (*p* = 0.0170), with an additional trend for higher LSf (*p* = 0.0648). For all other muscles and capillary parameters, HbA1c showed no significant associations (all *p* ≥ 0.13). Inclusion of group × age interaction terms or quadratic age^2^ terms did not improve model fit and did not change the LVf difference between groups, confirming that the lower LVf in DIA in T2DM was not explained by age distribution.

### Within-subject hierarchy among muscles

Because all four muscles were obtained from the same individuals, paired comparisons enabled analysis of intrinsic structural hierarchy independent of interindividual variability. Across subjects, LVf followed the order DIA > SC ≈ EXT > VL, while fibre diameter showed a contrasting pattern (VL > DIA > EXT > SC; all paired comparisons *p* < 0.01). No significant group-by-muscle interactions were detected, indicating that the relative ordering of capillary supply and fibre morphology across these muscles was preserved in T2DM.

## Discussion

This study analysed four functionally distinct skeletal muscles, SC, DIA, EXT and VL, obtained from the same older men with and without T2DM, using MyHC-based fibre typing, Sudan Black B IMCL staining and 3D confocal capillary morphometry. The T2DM group had HbA1c values generally within commonly targeted ranges and no documented advanced diabetic complications, supporting interpretation of the findings as early or moderate structural differences associated with diabetes and adiposity rather than overt advanced myopathy.

Overall, group differences were modest and muscle-specific, superimposed on a preserved hierarchy across muscles. Fibre-type composition and mean fibre diameter were largely maintained; IMCL was higher in SC and EXT; oxidative fibres (types 1 and 2a) contained more IMCL than type 2x fibres in both groups; and LVf was selectively reduced in the DIA, while other capillary indices were largely similar between groups. Among capillary endpoints, the DIA showed the clearest group difference, a selective reduction in LVf. SC showed the clearest fibre-size difference (larger type 1 fibres), EXT exhibited a small change in anisotropy and VL showed a modest increase in 2a/2x hybrids together with BMI-associated IMCL variation. Together, the pattern of results is consistent with a dominant influence of muscle-specific functional demands and oxidative phenotype, and with adiposity as an important covariate, rather than a uniform diabetes effect across the musculature, consistent with previous morphological and microvascular studies in diabetic and obese muscle (Umek et al. 2019).

The preservation of fibre-type composition in the postural (SC) and respiratory (DIA, EXT) muscles, and the modest increase in 2a/2x hybrids in VL, contrasts with reports of a more pronounced shift toward faster, more glycolytic phenotypes in some locomotor muscles in obesity and T2DM (Park et al. 2009; Andreassen et al. 2014). The larger SC type 1 fibres in T2DM may reflect chronic loading associated with higher body mass and sustained postural activity, although causal inference is limited by the cross-sectional, post-mortem design. Similar preservation or mild hypertrophy of fibres in frequently recruited muscles has been reported in models of obesity-induced insulin resistance, where increased mechanical load supports fibre growth despite systemic metabolic stress (Ato et al. 2019; Umek et al. 2021a).

Continuously active postural and respiratory muscles are exposed to sustained mechanical and metabolic demand, which can favour anabolic signalling and mitochondrial maintenance and may oppose atrophy programmes mediated by FoxO transcription factors (Sandri et al. 2006; Ogasawara et al. 2013). Conversely, intermittently recruited muscles, such as VL, may be more sensitive to reductions in habitual activity, particularly in older individuals. The fibre-type-specific age associations observed in DIA, EXT and VL should be interpreted as cohort-specific and may reflect selective survival of larger fibres, unmeasured activity differences or other confounding, rather than a general ageing signature. The minimal influence of BMI and HbA1c on fibre size suggest that, in this cohort of older men, muscle-specific factors, likely related to regional workload and oxygen demand, outweigh systemic factors such as BMI in determining fibre size (Frontera et al. 2000; Cameron et al. 2023). Collectively, these findings indicate that diabetes does not uniformly impair myofibre morphology; continuously active muscles, such as the postural SC, retain or even modestly increase their fibre calibre, which may reflect functional adaptation to sustained contractile and metabolic demands rather than pathological hypertrophy.

IMCL was modestly higher in T2DM, most clearly in SC and EXT, indicating that habitual postural or respiratory activity does not preclude greater lipid storage when adiposity is higher. This is consistent with evidence that IMCL content reflects both oxidative phenotype and lipid availability, and that IMCL per se is not a direct surrogate of insulin sensitivity without information on lipid species, subcellular localisation and turnover (Krssak et al. 1999; Goodpaster et al. 2001; Dubé et al. 2008; Coen and Goodpaster 2012). In the present study, IMCL was quantified as the percentage of fibre area occupied by Sudan Black B-positive lipid droplets, so this readout reflects bulk intracellular lipid staining on two-dimensional sections rather than lipid composition or subcellular droplet organisation. It does not distinguish lipid species, droplet size or subsarcolemmal from intermyofibrillar localisation. This limits mechanistic inference because ultrastructural studies in human skeletal muscle indicate that insulin resistance in T2DM is more closely related to compartment-specific lipid droplet remodelling, particularly expansion of subsarcolemmal lipid droplets, than to total IMCL alone (Nielsen et al. 2010, 2017; de Almeida et al. 2023). Accordingly, our Sudan Black B data should be interpreted as a comparative morphometric index of relative intracellular lipid storage, rather than a direct readout of lipotoxic lipid species or the subcellular lipid phenotype most closely linked to insulin resistance. These data reinforce the context dependence of IMCL, with muscle and fibre type shaping lipid storage patterns and adiposity contributing substantially to between-group differences (Goodpaster et al. 2001). The lack of clear group separation in the DIA and VL, together with the small, muscle-specific but fibre-type-independent changes, indicates that diabetes does not cause a uniform lipid overload across the musculature, but is superimposed on an existing gradient in which oxidative fibres store more lipid than glycolytic and hybrid fibres. The strong influence of BMI and the overall weak and regionally limited effects of HbA1c (with only a small isolated negative association in DIA type 1/2a fibres) support the view that adiposity and local oxidative propensity are the main drivers of IMCL in this cohort. Our BMI-adjusted models help account for group differences in adiposity, but they do not fully eliminate confounding by adiposity because the T2DM cohort had a substantially higher BMI and the study did not include a BMI-matched non-diabetic comparison group. This is particularly relevant for IMCL interpretation, as intramyocellular lipid accumulation is strongly influenced by adiposity and body fat status in human skeletal muscle. Therefore, the observed group differences may be interpreted as T2DM-associated alterations in the context of greater adiposity (Goodpaster et al. 2000; Moro et al. 2009). The slight negative association between age and IMCL in DIA and EXT may reflect reduced storage capacity or a shift towards greater lipid utilisation in chronically active respiratory tissues.

The 3D capillary analyses address limitations inherent to two-dimensional capillary indices. Two-dimensional measures derived from thin sections can be sensitive to fibre size, section orientation and regional sampling, which likely contributes to heterogeneous findings in human obesity and T2DM. Conventional stereological analyses have reported reduced capillarisation, unchanged indices or subtle changes that are difficult to interpret (Groen et al. 2014; Mortensen et al. 2019). In contrast, our 3D approach, combining confocal imaging, axial calibration, skeletonisation and vector-based reconstruction (Čebašek et al. 2010; Janáček et al. 2011), showed that microvascular architecture was largely preserved, with similar LVm, LL, LSf, MeanCap, branching density, and tortuosity between groups, and significant differences limited to DIA LVf and EXT anisotropy. A methodological limitation is that depth-uniform antibody penetration and fluorescence signal preservation were not quantitatively re-validated across the full 100 µm z-stack in the present dataset. However, our analysis was based on the established 3D confocal method of (Janáček et al. 2011), which identified penetration of fluorescent markers throughout thick human skeletal muscle sections as a required condition for reliable 3D capillary analysis and documented full-thickness penetration of the F8 endothelial antibody in originally 100-µm sections under optimised staining conditions. In the present study, all samples were processed at identical section thickness and with the same staining and imaging workflow, and linear Z-compensation was used to reduce depth-related attenuation. Nevertheless, residual depth-dependent signal loss or incomplete probe penetration could still lead to underestimation of absolute capillary length and branching measures and should therefore be considered when interpreting absolute 3D capillary metrics. Because the same protocol was applied across all muscles and both groups, such technical effects are less likely to account for the overall pattern of largely preserved capillary geometry, although subtle muscle-specific differences in labelling efficiency cannot be completely excluded.

Selective alterations in capillary supply metrics have been described in experimental obesity and diabetes, although the underlying driver varies by model and can include changes in fibre size, capillary remodelling or both (Poole et al. 2013; Gomes et al. 2017; Umek et al. 2021b). The DIA-specific LVf reduction in our study, therefore, likely reflects a modest imbalance between capillary length and fibre volume in a chronically loaded, oxidative muscle rather than frank vessel loss or network disorganisation. Covariate analyses further showed age-related simplification of the capillary network (lower tortuosity in SC and VL, shorter LL in VL), a positive association between BMI and LVf/LSf in the DIA, and DIA-specific associations of HbA1c within the T2DM group (shorter LL, higher MeanCap, trend to higher LS). Together, these patterns suggest that age and adiposity modulate quantitative capillary supply, particularly in the DIA, without disrupting overall microvascular topology, and that the DIA LVf deficit is a stable diabetes-related feature across the studied age range.

Across both groups, the intrinsic structural hierarchy among muscles was preserved: LVf followed the order DIA > SC ≈ EXT > VL, whereas fibre diameter exhibited the inverse pattern. This reciprocal relationship reflects a consistent design principle in skeletal muscle, whereby smaller oxidative fibres are supplied by proportionally denser capillary networks to optimise oxygen diffusion (Wüst et al. 2009) and parallels previous observations in healthy human muscle (Pollak et al. 2025) and in experimental obesity and diabetes models (Umek et al. 2021a). Its persistence suggests that mechanisms linking myofibre structure and capillary architecture remain largely intact in this cohort. Functionally, such structural stability may contribute to the relative preservation of respiratory and postural performance in individuals with T2DM, even though microvascular impairments and endothelial dysfunction are well documented in other tissues (Groen et al. 2014; Sörensen et al. 2016).

Given the chronic nature of diabetic tissue remodelling, the duration of diabetes is an important contextual exposure variable for interpreting the modest, muscle-specific phenotypes observed here. In our cohort, the recorded time since diagnosis was long (mean duration 16.5 ± 5.1 years), indicating long-standing diagnosed T2DM and providing context for interpreting the modest, muscle-specific phenotypes observed here. However, duration derived from clinical diagnosis is an imprecise surrogate for true glycaemic exposure, because T2DM commonly remains clinically unrecognised for years after onset of dysglycaemia (Harris et al. 1992). Accordingly, any inference that links ‘time since diagnosis’ to cumulative hyperglycaemic burden (or to the magnitude of structural differences) should be made cautiously. In addition, all individuals with T2DM were managed with oral antihyperglycaemic therapy (monotherapy or combination therapy), which may itself influence skeletal muscle metabolic and microvascular endpoints; for example, metformin has been shown in insulin-resistant humans to improve insulin-mediated skeletal muscle microvascular responsiveness alongside improved muscle glucose disposal, raising the possibility that treatment could have attenuated or modified diabetes-associated microvascular and lipid-related signals in this relatively well-controlled cohort (Jahn et al. 2022).

The main strengths of this study are its autopsy-based design, enabling simultaneous sampling of four anatomically and functionally distinct muscles from the same individuals, and the combined assessment of fibre type, IMCL and 3D capillary architecture. This design reduces interindividual variability, a major confounder in biopsy-based research, and provides a structurally coherent view of how T2DM affects the muscular system. However, several limitations should be acknowledged. The cohort consisted solely of older male individuals, limiting generalisability to women and younger age groups, in whom sex hormones and physical activity patterns may influence muscle phenotype and vascularisation. The post-mortem, cross-sectional design precludes assessment of dynamic perfusion, mitochondrial function or muscle performance, and structural preservation therefore cannot be directly equated with preserved functional capacity (Eržen et al. 2018). Mitochondrial enzyme histochemistry was not performed because, in post-mortem muscle, interpretation of enzyme-based histochemical readouts would require dedicated validation and standardisation of post-mortem interval-related and tissue-handling effects across all four muscles, which was beyond the scope of the present study; accordingly, the present data do not directly address mitochondrial alterations reported in skeletal muscle in T2DM (Kelley et al. 2002). An additional limitation of the autopsy-based design is that agonal physiology cannot be fully controlled. Pre-mortem hypoxia and hemodynamic instability can affect endothelial barrier function and capillary permeability, and hypoxia can also alter skeletal muscle fatty acid handling and intracellular lipid composition (Morash et al. 2013). Post-mortem tissue studies also indicate that death-related and post-mortem factors may influence biological readouts in a tissue-specific manner (Ferreira et al. 2018). Although all samples were collected within 24 h post-mortem, and tissues were grossly well preserved, subtle autolytic or fixation-related effects cannot be fully excluded.

Furthermore, while cause-of-death data were available for all cases, agonal duration and peri-mortem treatment and medication data were not available in a standardised form, and cause-of-death profiles were not matched between groups. Therefore, the IMCL and microvascular findings should be interpreted as muscle-specific alterations observed in well-characterised autopsy cohorts, in which diabetes-related remodelling may be accompanied or modulated by agonal and peri-mortem factors. The T2DM group was also not BMI-matched to controls, so despite statistical adjustment for BMI, residual confounding by adiposity cannot be excluded, especially for IMCL-related outcomes and potentially for microvascular phenotypes linked to obesity-associated metabolic dysfunction. Finally, molecular markers of angiogenesis, oxidative metabolism and inflammation (e.g. VEGF, PGC-1α, TNFα) were not measured, so mechanistic interpretations remain hypothesis-generating.

## Conclusions

In this cohort of older men, T2DM was associated with muscle-specific, not generalised, structural differences across functionally diverse skeletal muscles. Fibre-type composition and mean fibre diameter were largely preserved, with larger type 1 fibres in SC and a modest increase in 2a/2x hybrid fibres in VL. IMCL was modestly higher in the SC and EXT, whereas VL and DIA showed no significant group differences, and oxidative fibres contained more IMCL than type 2x fibres in both groups. Three-dimensional capillary morphometry showed a selective reduction in DIA LVf and a small increase in anisotropy in EXT, with otherwise preserved capillary geometry and maintenance of the cross-muscle hierarchy linking fibre calibre and capillary supply. Across outcomes, adiposity and age showed stronger and more consistent associations than HbA1c. BMI emerged as the strongest predictor of IMCL and DIA capillary supply, whereas age influenced fibre size and capillary path geometry. Taken together, these findings indicate that local functional demand, oxidative phenotype, adiposity and ageing are important contributors to long-term muscle architecture in this autopsy cohort, although interpretation of the observed differences should take early post-mortem factors into account. The relative structural preservation of postural and respiratory muscles may help explain the absence of pronounced functional deficits in everyday activities despite systemic metabolic dysfunction. Future work integrating 3D microvascular morphometry with lipid species profiling, perfusion imaging, mitochondrial assessments and functional testing in the same individuals will be required to link these subtle structural differences to physiological performance and to identify mechanisms supporting muscle microvascular resilience in T2DM.

## Supplementary Information

Below is the link to the electronic supplementary material.

Supplementary file1 (DOCX 17 KB)

## Data Availability

The datasets used and analysed during the present study are available from the corresponding author upon reasonable request.
