# Supplementary material for: Fibre morphology, intramyocellular lipid content and 3D capillary architecture in human postural, respiratory and locomotor muscles in type 2 diabetes mellitus
Source: Histochem Cell Biol. 2026 Apr 6;164(1):20. doi: 10.1007/s00418-026-02477-7 (PMC13053442; doi:10.1007/s00418-026-02477-7)
Supplement: Supplementary file 1 — Supplementary file1 (DOCX 17 KB) [file 418_2026_2477_MOESM1_ESM.docx]

**Fibre morphology, intramyocellular lipid content and 3D capillary architecture in human postural, respiratory and locomotor muscles in type 2 diabetes mellitus**

Authors

Nataša Pollak, Jiří Janáček, František Saudek, Erika Cvetko, Barbora Radochová, Armin Alibegović, Chiedozie Kenneth Ugwoke, Davide Alessandro Basello, Žiga Šink, Luka Pušnik, Rok Tit Tomazin, Nejc Umek

**Supplementary Table S1.** Average number of analysed fibres per muscle for each fibre type in control and T2DM groups

| **Muscle** | **Group** | **Type 1** | **1/2a hybrid** | **Type 2a** | **2a/2x hybrid** | **Type 2x** | **Total fibres analysed** |
| --- | --- | --- | --- | --- | --- | --- | --- |
| SC | Control | 63.88 ± 11.50 | 4.07 ± 2.40 | 18.32 ± 5.54 | 18.51 ± 4.62 | 11.06 ± 5.88 | 115.84 ± 14.50 |
| SC | T2DM | 62.76 ± 11.07 | 2.36 ± 1.29 | 14.22 ± 3.49 | 27.15 ± 6.45 | 9.99 ± 4.64 | 116.48 ± 15.83 |
| DIA | Control | 72.28 ± 13.01 | 3.66 ± 2.74 | 25.24 ± 9.15 | 11.75 ± 5.18 | 4.62 ± 3.70 | 117.55 ± 13.41 |
| DIA | T2DM | 72.66 ± 12.73 | 4.23 ± 1.70 | 23.96 ± 6.13 | 12.80 ± 4.37 | 2.07 ± 1.45 | 115.72 ± 15.57 |
| EXT | Control | 61.00 ± 10.98 | 3.19 ± 2.49 | 24.89 ± 6.31 | 16.25 ± 5.29 | 9.67 ± 6.49 | 115.00 ± 14.14 |
| EXT | T2DM | 62.90 ± 11.18 | 4.94 ± 2.45 | 28.26 ± 7.07 | 15.45 ± 4.84 | 6.51 ± 4.01 | 118.06 ± 16.48 |
| VL | Control | 48.27 ± 9.67 | 3.61 ± 2.01 | 34.42 ± 6.77 | 19.39 ± 5.77 | 11.89 ± 5.42 | 117.58 ± 16.55 |
| VL | T2DM | 42.43 ± 7.51 | 3.49 ± 1.89 | 26.78 ± 6.39 | 30.15 ± 6.29 | 13.07 ± 3.99 | 115.92 ± 15.46 |

Values are presented as mean ± SD fibres per muscle. Fibre typing, fibre diameter, and intramyocellular lipid (IMCL) quantification were performed on the same set of fibres. Total fibres analysed denotes the average total number of annotated fibres per muscle in each study group. SC, splenius capitis; DIA, diaphragm; EXT, external intercostal; VL, vastus lateralis; T2DM, type 2 diabetes mellitus.
